# Immune cell profiles in synovial fluid after anterior cruciate ligament and meniscus injuries

**DOI:** 10.1186/s13075-021-02661-1

**Published:** 2021-11-04

**Authors:** Sophia Y. Kim-Wang, Abigail G. Holt, Alyssa M. McGowan, Stephanie T. Danyluk, Adam P. Goode, Brian C. Lau, Alison P. Toth, Jocelyn R. Wittstein, Louis E. DeFrate, John S. Yi, Amy L. McNulty

**Affiliations:** 1 Department of Biomedical Engineering, Duke University, Durham, NC USA; 2 Department of Orthopaedic Surgery, Duke University School of Medicine, Durham, NC USA; 3 Department of Surgery, Duke University School of Medicine, Durham, NC USA; 4 Department of Mechanical Engineering and Materials Science, Duke University, Durham, NC USA; 5 Department of Pathology, Duke University School of Medicine, Durham, NC USA

**Keywords:** Monocytes, Macrophages, Cartilage, B cells

## Abstract

**Background:**

Anterior cruciate ligament (ACL) and meniscus tears are common knee injuries. Despite the high rate of post-traumatic osteoarthritis (PTOA) following these injuries, the contributing factors remain unclear. In this study, we characterized the immune cell profiles of normal and injured joints at the time of ACL and meniscal surgeries.

**Methods:**

Twenty-nine patients (14 meniscus-injured and 15 ACL-injured) undergoing ACL and/or meniscus surgery but with a normal contralateral knee were recruited. During surgery, synovial fluid was aspirated from both normal and injured knees. Synovial fluid cells were pelleted, washed, and stained with an antibody cocktail consisting of fluorescent antibodies for cell surface proteins. Analysis of immune cells in the synovial fluid was performed by polychromatic flow cytometry. A broad spectrum immune cell panel was used in the first 10 subjects. Based on these results, a T cell-specific panel was used in the subsequent 19 subjects.

**Results:**

Using the broad spectrum immune cell panel, we detected significantly more total viable cells and CD3 T cells in the injured compared to the paired normal knees. In addition, there were significantly more injured knees with T cells above a 500-cell threshold. Within the injured knees, CD4 and CD8 T cells were able to be differentiated into subsets. The frequency of total CD4 T cells was significantly different among injury types, but no statistical differences were detected among CD4 and CD8 T cell subsets by injury type.

**Conclusions:**

Our findings provide foundational data showing that ACL and meniscus injuries induce an immune cell-rich microenvironment that consists primarily of T cells with multiple T helper phenotypes. Future studies investigating the relationship between immune cells and joint degeneration may provide an enhanced understanding of the pathophysiology of PTOA following joint injury.

## Background

Anterior cruciate ligament (ACL) ruptures and meniscal tears are common among athletes, and frequently occur in the general population [1]. In the USA, over 400,000 ACL injuries occur annually [2], and more than 500,000 meniscus surgeries were performed in 2014 [3]. Moreover, the long-term sequelae of both ACL and meniscal injuries include pain, joint instability, and post-traumatic osteoarthritis (PTOA) in approximately 50% of patients [1, 4–7].

Despite the high rate of PTOA following joint injury, the factors that contribute to PTOA development remain unclear. Several studies have investigated altered knee biomechanics after ACL [8–13] and meniscus injuries [14–18], suggesting possible associations with PTOA. Other studies suggest that biological changes [19–24] that occur within the joint following injury may play a role in PTOA development as well. Nonetheless, there is little data on the biochemical and cellular environment of the joint following injury.

A few studies have measured biochemical and gene expression changes following joint injury. In the synovial fluid of ACL-injured patients, cytokines and catabolic biomarkers, including interleukin (IL)-1α, IL-1β, IL-6, IL-8, lubricin, tumor necrosis factor (TNF)-α, matrix metalloproteinases (MMPs), and cartilage oligomeric matrix protein (COMP), are increased in comparison to concentrations found in the synovial fluid of healthy, uninjured subjects [19–21, 25–27]. Among meniscus-injured subjects, increases in prostaglandin E2 (PGE2), MMP activity, MMP-3, IL-6, monocyte chemotactic protein-1, and macrophage inhibitory protein (MIP)-1β have been detected in the synovial fluid of injured joints [16, 20, 23, 28]. This work has identified cytokines, catabolic biomarkers, and chemokines that are altered in injured joints and may contribute to PTOA development. However, the specific cell types in the synovial fluid that mediate the production of cytokines and chemokines have not been studied in ACL and meniscus-injured patients.

Only a few studies have evaluated cell types that are altered following joint injury in animal models. Specifically, recent animal models have shown that macrophages may be involved in the progression of PTOA after meniscus injury [29, 30]. In addition, several studies have evaluated immune cell profiles in patients with osteoarthritis (OA) and rheumatoid arthritis (RA). In patients diagnosed with primary OA, studies have shown an increased presence of activated macrophages and T cells with a higher CD4/CD8 ratio of T cells in the serum, synovial fluid, and synovial tissues [22, 31–35]. RA has also been well-studied with regard to subsets of T cells, demonstrating higher percentages of CD4 T cells in the peripheral blood and synovial membrane compared to OA and control subjects [33, 36–38]. However, there is a lack of data on immune cell profiles in the synovial fluid of joints with ACL or meniscal injuries. Therefore, in this pilot study, we aimed (1) to determine which immune cell subsets were present in the synovial fluid following joint injury, and based on this profile (2) to identify the specific CD4 and CD8 T cell subset(s) that migrate to the injured synovial fluid.

## Methods

### Inclusion criteria

All study procedures and protocols were approved by the Institutional Review Board at Duke University School of Medicine. Patients undergoing ACL reconstruction and/or meniscus repair or meniscectomy were enrolled in the study. The following inclusion criteria were also used: minimum of 12 years of age; BMI between 18.5 and 30.0 kg/m^2^; no history of diagnosed arthritis; and a non-operative contralateral (normal) knee with no history of knee injury or surgery. A total of 29 subjects (14 meniscus-injured and 15 ACL-injured) that met the inclusion criteria were consented. Of the 15 ACL-injured, 5 had a concomitant meniscus injury, which will be referred to as “ACL+meniscus.”

### Synovial fluid collection and cell isolation

All study activities occurred on the day of surgery. In the operating room prior to incision, synovial fluid was aspirated from both the injured and normal knees. If necessary to obtain fluid, the surgeon lavaged the joint with 1–20 mL of normal saline. Lavage was necessary in 28 out of 29 normal knees and 5 out of 29 injured knees. Once aspirated, the synovial fluid was transferred to 15 mL conical tubes containing protease inhibitor (Millipore Sigma, Burlington, MA) and placed on ice. Tubes were spun at 350*g* for 10 min at 4 °C to pellet the cells. The synovial fluid supernatant was removed and frozen. Next, the entire cell pellet was resuspended with gentle vortexing and the red blood cells were lysed by adding lysing solution (BD Biosciences, San Jose, CA) for 3 min. Then, the cells were centrifuged and resuspended for cell surface staining.

### Flow cytometry

Analysis of immune cells in the synovial fluid was performed by polychromatic flow cytometry (PFC) based on published gating strategies [39, 40]. Cells were first incubated with a Zombie dye for 15 min at room temperature to detect dying cells. Cells were then washed with PBS + 2% FBS (FACS wash). Next, cells were incubated with Fc block (BD Biosciences) for 15 min at 4 °C and washed with FACS wash. Surface staining was performed with an antibody cocktail consisting of fluorescent antibodies against cell surface proteins. Cells were stained for 25 min in the dark at 4 °C, and unbound antibodies were washed out by centrifugation. Lastly, cells were fixed with 1% paraformaldehyde prior to acquisition on a Symphony X50 flow cytometer (BD Biosciences), and data were analyzed using Flowjo software (BD Biosciences). All events from each stained sample were acquired by flow cytometry.

The antibodies and viability dyes used for the broad spectrum immune cell panel and T cell panel are listed in Tables 1 and 2, respectively.

**Table 1:** Antibodies and dyes used for the broad spectrum immune cell panel

Broad spectrum panel				
Marker	Clone	Fluorophore	Dilution	Vendor
Zombie viability dye	N/A	nIR	1:100	Biolegend (San Diego, CA)
CD3	SK7	AlexaFluor 700	1:10	Biolegend
CD14	M5E2	PB	1:10	BD Biosciences (San Jose, CA)
CD16	3G8	APC	1:10	Biolegend
CD19	HIB19	PE-Cy5	1:10	Biolegend
CD45	HI30	PE	1:10	Sony (San Jose, CA)
CD86	FUN-1	BV510	1:20	BD Biosciences
HLA-DR	L243	BV605	1:20	Biolegend

**Table 2:** Antibodies and dyes used for the T cell panel

T cell panel				
Marker	Clone	Fluorophore	Dilution	Vendor
Zombie viability dye	N/A	Aqua	1:100	Biolegend (San Diego, CA)
CD3	SK7	AlexaFluor 700	1:20	Biolegend
CD4	SK3	BUV805	1:20	BD Biosciences (San Jose, CA)
CD8	SK1	APC-Cy7	1:10	Biolegend
CD14	MOP9	BUV395	1:20	BD Biosciences
CD25	2A3	BB515	1:40	BD Biosciences
CD38	HIT2	BV421	1:20	Biolegend
CD45	HI30	PE-Cy5	1:20	Biolegend
CD45RA	HI100	PerCP-Cy5.5	1:10	Biolegend
CD127	A019D5	BV650	1:20	Biolegend
CCR4	L291H4	BV605	1:20	Biolegend
CCR6	G034E3	BV785	1:20	Biolegend
CCR7	150503	PE-CF594	1:20	BD Biosciences
CCR10	314305	APC	1:20	R&D Systems (Minneapolis, MN)
CXCR3	1C6/CXCR3	PE	1:2.5	BD Biosciences
CXCR5	J252D4	PE-Cy7	1:78	Biolegend
HLA-DR	G46-6	BUV661	1:313	BD Biosciences

### Statistical analyses

Demographics (age and sex) and clinical characteristics (time from injury, medical diagnosis, and cause of injury) were described for each participant for both the broad spectrum immune panel and the T cell-specific panel. We used Shapiro-Wilk tests to assess the normality of all count, percentage, and normalized data. Due to some cell percentages and counts violating normality assumptions, non-parametric Wilcoxon signed-rank tests were used to analyze differences between paired normal and injured knees for the broad panel cell counts and for the T cell panel normalized data. For the T cell-specific panel, CD3 cell counts were normalized to the leukocyte (CD45) count in the same knee, capturing the proportion of CD3 cells per all viable immune cells. In order to ensure enough cells for subsequent analyses in the T cell panel, a T cell (CD3) count threshold was set at 500 cells. A Fisher’s exact test was used to assess the frequency of a T cell count greater than 500 cells in both the normal and injured knee synovial fluid samples. Non-parametric one-way ANOVAs and Mann-Whitney-Wilcoxon post hoc tests were used for the injured only to determine differences in T cell percentage, age, and time from injury comparisons across injury types. All statistical analyses were performed using JMP (SAS Institute Inc., Cary, NC). Significance was set at an alpha of *p* < 0.05.

## Results

### Assessment of immune cell subsets in the synovial fluid

**Fig. 1:**
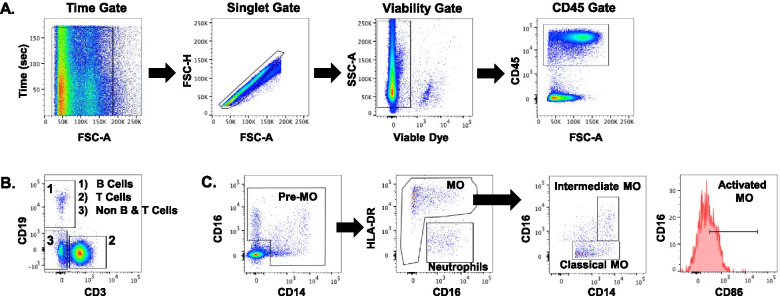
Representative flow cytometry gating strategy for the broad spectrum panel. **A** To identify immune subsets, non-relevant events including debris, doublets, red blood cells, and dead cells were removed through gating using the Flowjo software. Gating on CD45 cells, **B** immune cells were then segregated into (1) B cells, (2) T cells, and (3) innate immune cells (non B and T cells). **C** Gating on the innate population from **B**3, cells were divided into neutrophils and monocyte (MO) subsets: classical, intermediate, and activated, which were gated off of total monocytes

**Fig. 2:**
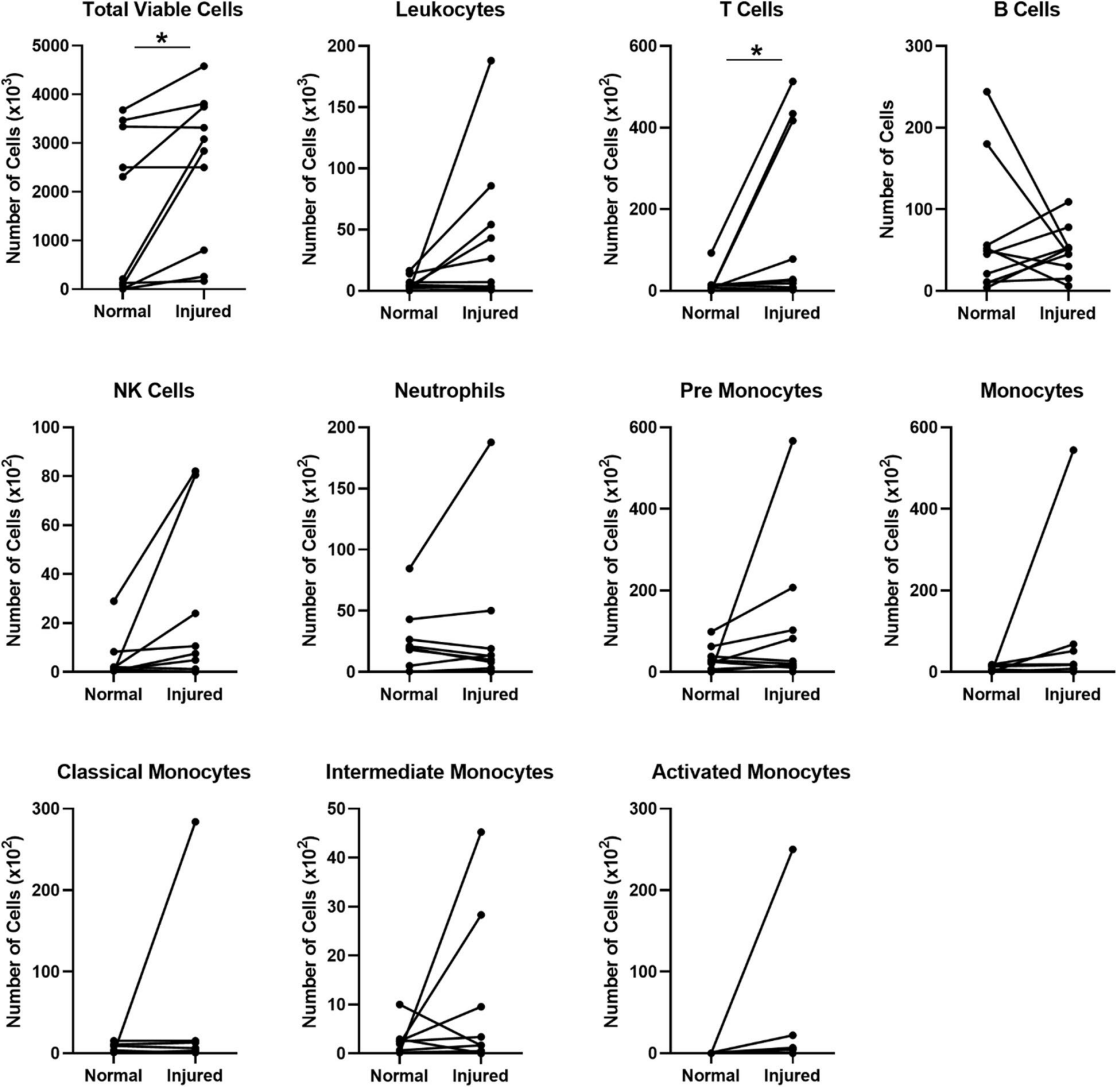
Broad spectrum immune panel cell counts in the aspirated synovial fluid of normal and injured knees. Individual data points for each patient are presented with lines between paired samples (*N* = 10). *p < 0.05

Using a broad spectrum immune cell flow cytometry panel, we analyzed synovial fluid from 10 subjects (mean age: 25.0 ± 4.6 years). Of these subjects, 3 had isolated meniscal tears, 5 had isolated ACL tears, and 2 had concomitant ACL+meniscus tears. Subject demographics are listed in Table 3. Figure 1 shows a representative gating scheme for the broad spectrum analysis. Within the synovial fluid, we were able to detect innate and adaptive immune cells, including B cells, T cells, monocytes, dendritic cells, and natural killer (NK) cells. Total viable cells were significantly increased in the injured knees as compared to the normal knees (Fig. 2, *p* < 0.05). However, there was no significant difference in the percentage of viable cells in the normal (median: 99.5%) and injured knees (median: 99.5%). In comparison to normal knees, the median number of leukocytes (CD45) was elevated nearly 4-fold in the injured synovial fluid (Fig. 2, *p* = 0.06). T cells (CD3) were significantly increased in the injured knees (*p* < 0.05). While not statistically significant, on average there were higher numbers of NK cells (*p* = 0.08), neutrophils (*p* = 1.0), pre-monocytes (*p* = 0.11), monocytes (*p* = 0.49), classical monocytes (*p* = 0.57), intermediate monocytes (*p* = 0.28), and activated monocytes (*p* = 0.19) detected in the synovial fluid of injured knees than the normal knees. Based on the significant difference in the number of T cells between normal and injured knees and the large percentage of T cells in the joints (Fig. 1B), we focused subsequent analyses on immune profiling subsets within the T cell population.

**Table 3:** Demographics of subjects evaluated with the broad spectrum immune panel

Broad spectrum panel subject demographics				
Sex	Age	Time from injury	Diagnosis	Cause of injury
F	22	3 years	Meniscus	Snowboarding
M	20	23 days	ACL+meniscus	Soccer
M	32	51 days	Meniscus	Treadmill running
F	18	52 days	ACL	Soccer
F	30	6 years	Meniscus	Non-specific
M	21	455 days	ACL	Soccer
M	27	91 days	ACL	“Jumping in a mosh pit”
F	28	53 days	ACL	Soccer
M	25	38 days	ACL	Basketball
M	27	37 days	ACL+meniscus	Frisbee golf

### Delineation of synovial fluid T cell subsets

**Fig. 3:**
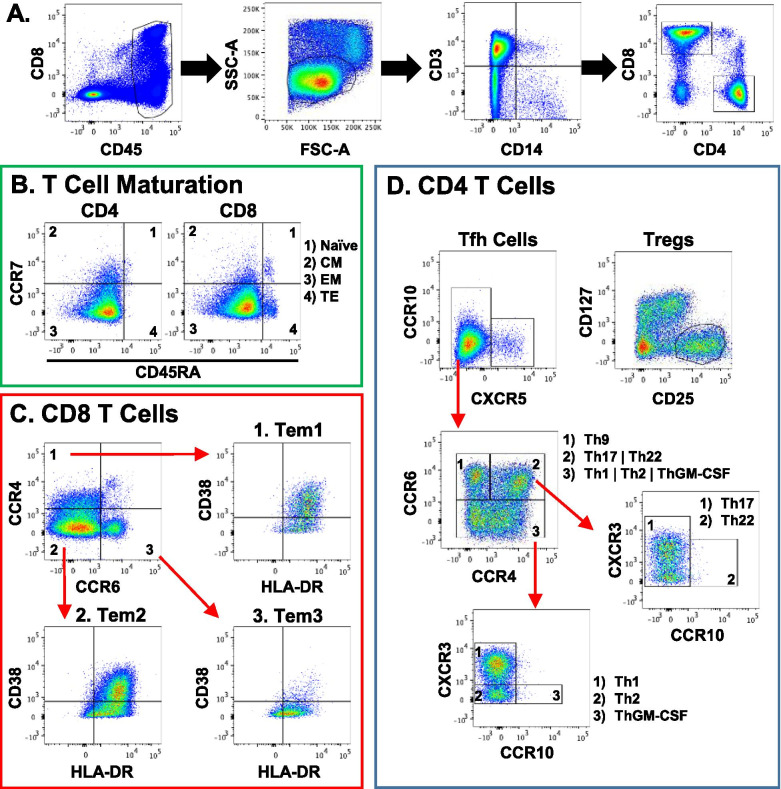
Immune profiling synovial fluid-derived CD4 and CD8 T cells. **A** Gating strategy to exclude debris, dead cells, and non-CD3 T cells to visualize CD4 and CD8 T cells. **B** Maturation profile of CD4 and CD8 T cells in the synovial fluid (CM: central memory, EM: effector memory, TE: terminal effectors). **C** Division of activated CD8 T cells into Tem1, Tem2, and Tem3 subsets. **D** Visualization of differentiated CD4 Th subsets using chemokine receptors. The high dimensional panel identifies Th1, Th2, Th9, Th17, Th22, ThGM-CSF, Tfh, and Treg subsets

**Fig. 4:**
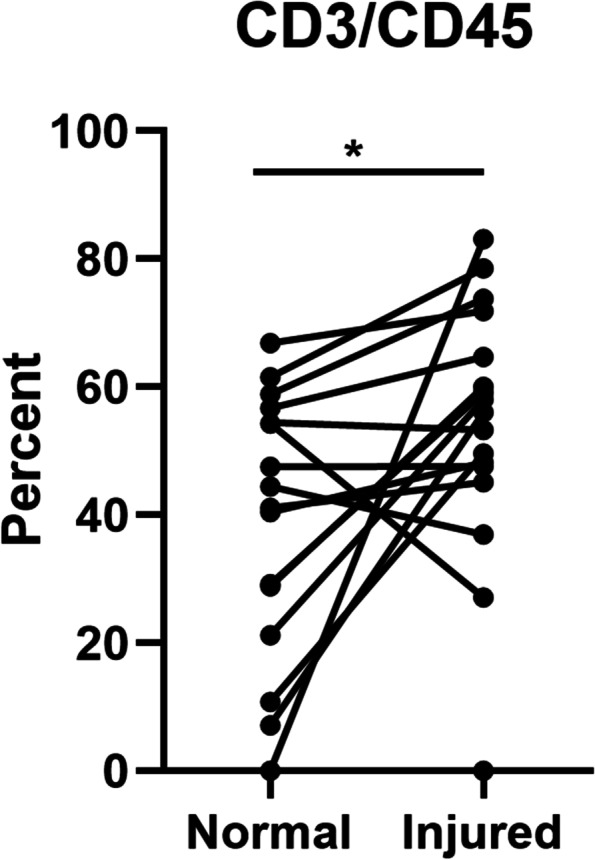
T cells (CD3) normalized to leukocytes (CD45) using the T cell-specific panel (*N* = 17). Individual data points for each patient are presented with lines between paired samples. *p < 0.05

In a separate group of 19 subjects (mean age: 33.7 ± 11.1 years) with ACL and/or meniscus tears (Table 4), we focused our analysis on identifying T cell subsets that are recruited to the synovial fluid following knee injury. Of these subjects, there were 11 isolated meniscal tears, 5 isolated ACL tears, and 3 concomitant ACL+meniscus tears. Figure 3 shows a representative gating scheme for the T cell profiling analysis. In order to ensure enough cells for subsequent analyses, a T cell (CD3) count threshold was set to 500 cells. For normal knee synovial fluid samples, only 4 out of 19 samples had more than 500 T cells, while 17 of 19 injured synovial fluid samples met this threshold. Overall a significantly lower percentage of normal knees (21%) met this threshold compared to injured knees (89%) (Table 5, *p* < 0.0001). To account for dilution effects from the normal saline lavage during aspiration, we normalized the T cell count (CD3) to total leukocytes (CD45). Normalized T cell counts were also significantly increased in injured knees as compared to normal knees (Fig. 4, *p* < 0.05).

**Table 4:** Demographics of subjects analyzed with the T cell-specific panel

T cell panel subject demographics				
Sex	Age	Time from injury	Diagnosis	Cause of injury
F	40	3 months	ACL	Slipped on wet floor
M	19	6 weeks	ACL+meniscus	Football
F	49	1 month	ACL	Football
M	28	4 months	ACL	Soccer
F	34	3 months	ACL	Running
M	20	3 months	ACL+meniscus	Basketball
F	18	1 month	ACL+meniscus	Basketball
M	30	2 months	ACL	Soccer
M	37	2 months	Meniscus	Non-specific
M	53	2 months	Meniscus	Non-specific
M	48	6 months	Meniscus	Non-specific
M	43	chronic	Meniscus	Non-specific
F	39	1 year	Meniscus	Non-specific
M	45	5 months	Meniscus	Soccer
M	27	4 months	Meniscus	Running
F	21	6 months	Meniscus	Soccer
F	34	1 year	Meniscus	Running
M	19	1 month	Meniscus	Running
M	36	1 year	Meniscus	Non-specific

**Table 5:** Contingency table of synovial fluid samples meeting the T cell threshold (Fisher’s exact test (2-tail): *p < 0.0001)

T cells ≥ 500			
	No	Yes	Total
**Normal**	15	4	19
**Injured**	2	17	19
Total	17	21	38

**Fig. 5:**
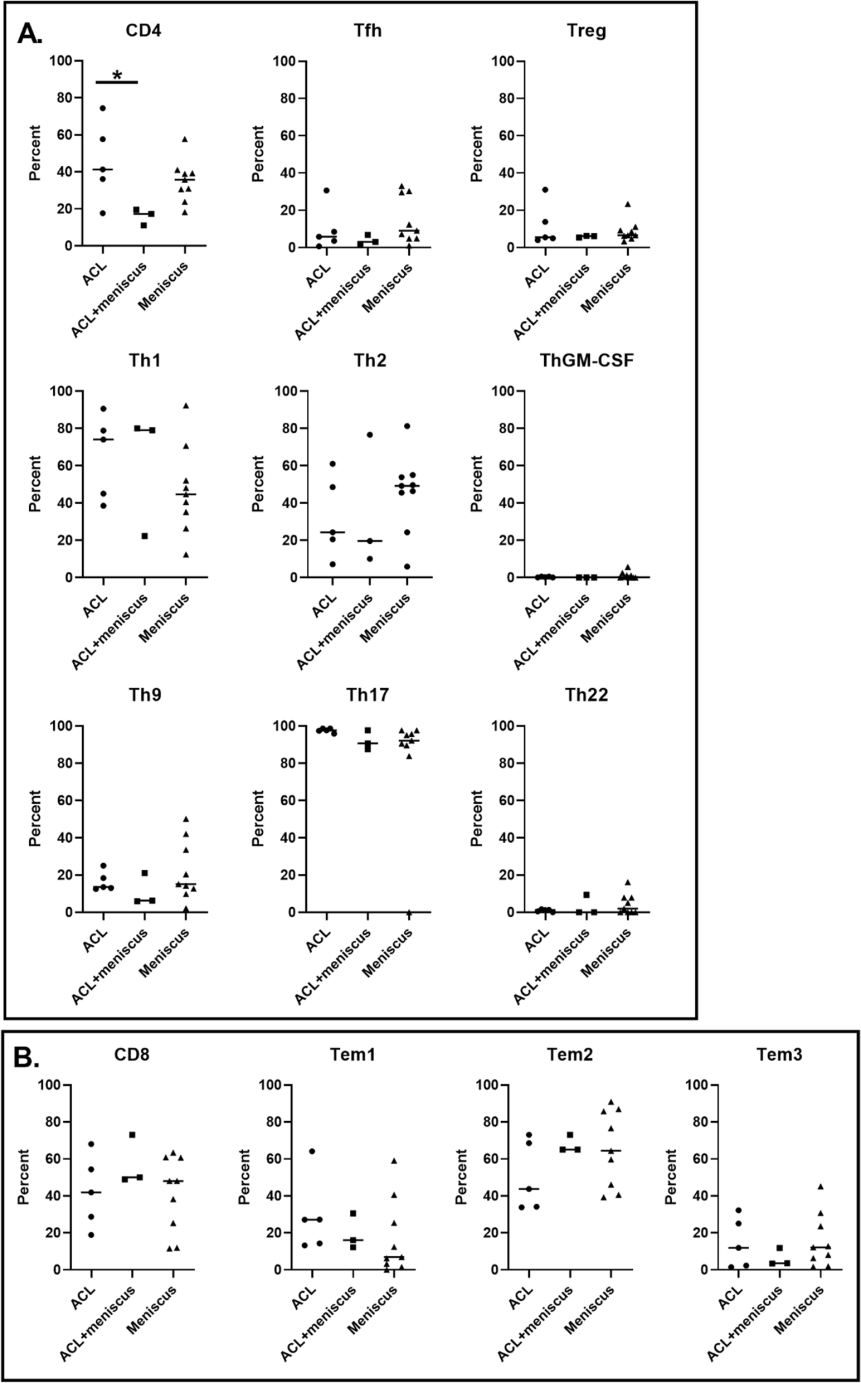
T cell panel results by injury type. **A** CD4 T cells and CD4 subset (Tfh, Treg, Th1, Th2, ThGM-CSF, Th9, Th17, and Th22) frequencies (individual data points and median) in the synovial fluid of injured knees. A statistically significant difference was detected in the percentage of CD4 T cells between ACL-injured and ACL+meniscus-injured knees (*p = 0.042). **B** CD8 T cells and CD8 subset (Tem1, Tem2, and Tem3) frequencies in the synovial fluid of injured knees. No statistically significant differences were detected (*p* > 0.05) (*N* = 17 injured samples with 500 or more T cells)

Among the 17 injured samples that met the T cell threshold, a significant difference in age was detected between injury groups (*p* = 0.047). In particular, there was a significant difference in age when comparing the concomitant ACL+meniscus group (19 years) to the isolated ACL (36 years, *p* < 0.05) and isolated meniscus (35 years, *p* < 0.05) groups. No significant differences were detected in the time from injury to surgery among injury groups. In-depth analysis of T cells revealed a heterogeneity of T cell subsets within the synovial fluid of both ACL- and meniscus-injured knees (Fig. 3). The majority of the CD4 and CD8 T cells were effector-memory cells based on their maturation profile (Fig. 3B). Using a combination of chemokine receptors, CD4 T cells (also known as T helper cells) were broken down into T helper subsets: Th1, Th2, Th9, Th17, Th22, ThGM-CSF, follicular helper cells (Tfh), and regulatory T cells (Tregs) (Fig. 5A). When analyzed by injury type, a significant difference in the overall percentage of CD4 T cells was detected (*p* = 0.042), with ACL-injured knees having a higher percentage of CD4 T cells than ACL+meniscus-injured knees. No significant differences were detected among injury types for T helper subsets. However, among these subsets, Th1, Th2, and Th17 were on average higher in the injured knees. The injured synovial fluid samples also contained CD8 T cells (also known as cytotoxic T cells) and the following subsets of CD8 T cells: T effector memory (Tem) 1, Tem2, and Tem3 (Fig. 5B). However, there were no significant differences in the CD8 T cell subsets by injury type.

## Discussion

The overall goal of the present study was to identify the major immune cell subsets in the synovial fluid of patients with ACL and/or meniscus injuries. Notably, more immune cells were found in injured knees compared to their paired normal knees. In our initial studies surveying innate and adaptive cell subsets in ACL- and meniscus-injured knees, we observed that T cells were elevated in the injured knees. Within the injured knees, we were able to differentiate CD4 and CD8 T cells into subsets and detected a significantly higher percentage of CD4 T cells in ACL- than ACL+meniscus-injured knees. However, we did not detect significant differences in T cell subsets across injury type. Collectively, our results provide foundational data showing that ACL and meniscus injury induce an immune cell-rich microenvironment that is heterogeneous, consisting primarily of T cells with multiple Th phenotypes. These phenotypes are similar to Th cells found in subjects with advanced OA and RA [33, 35, 41], suggesting that these immune cells may be important mediators of joint changes after ACL and meniscus injuries that contribute to PTOA development.

In the broad immune cell panel, we observed statistically significant increases in total viable cells and T cells in the injured knees. Additionally, while not statistically significant, we observed greater median counts in the injured knees for all immune cell types, except B cells and neutrophils. In this study, we hypothesized that immune cells would be elevated following ACL and meniscus injury based on previous studies of symptomatic knee OA [22]. In particular, prior work has shown increased macrophages and monocytes in patients with symptomatic OA [22]. Monocytes and macrophages can produce MIP-1, interferon (IFN)-γ, IL-1β, and MMPs, which have been shown to be increased after ACL [21, 28] and meniscus injuries [20, 23, 28]. However, in the present study, we found significant increases in T cells in ACL- and meniscus-injured subjects. In the inflamed synovium of RA patients, T cells in close proximity to macrophages induce cytokine production [42] either via direct cell interactions [43] or through the production of cytokines, such as IL-17 and IFN-γ [44]. While prior studies have not definitively established T cells as mediators of OA [45, 46], both IL-17 [41] and IFN- γ [47] are known to stimulate other cells, cytokines, and chemokines that lead to inflammation and destruction of bone and cartilage. To this point, in an ACL transection mouse model of OA, CD4 T cells increased early after injury and induced MIP-1γ production in the synovium, causing more cartilage degeneration [48]. Thus, our findings along with previous studies [41–48] suggest that the rise in T cells may occur downstream of increases in monocytes and macrophages. However, collection of synovial fluid at additional time points will be necessary to fully characterize the time course of immune cell changes following joint injury. Additionally, future studies investigating the relationship between macrophages, monocytes, and T cells may provide an enhanced understanding of the pathophysiology of PTOA following joint injury.

Our findings represent a snapshot of the immune cell profile in the synovial fluid of the joint. Different joint tissues, such as the synovium, cartilage, and menisci, may have different immune cell profiles. For example, among human subjects with acute articular ankle fracture, ankle OA, and knee OA, there was an increase in macrophages in the synovium of acute articular ankle fracture subjects [49]. Also, in the synovium of dogs with cranial cruciate ligament tears, CD4, CD8, and non-CD4/CD8 T cells were increased compared to healthy dogs [50]. Furthermore, in menisci of human subjects with RA and OA, immunohistochemistry showed that macrophages, T cells, and B cells were increased in the outer, vascular region compared to the less vascular, central region of the tissue [51]. Similarly, a study investigating macrophages and T cells in a mouse model of ACL transection found increased staining of macrophages and T cells in the vascularized region of the menisci [52]. These increases in specific immune cells of different tissues indicate the importance of localized profiling of cell types to study progression of PTOA.

The overall percentage of CD4 T cells differed significantly among injury types, with ACL+meniscus and ACL groups having the lowest and highest percentages, respectively. Prior work has shown that there is an increase in the number of CD4 T regulatory cells that occurs with aging [53]. Therefore, the significant difference in CD4 T cells between the concomitant ACL+meniscus and isolated ACL and meniscus groups could be due to age. However, a variety of factors, such as age, sex, and time from injury, may influence these results as well. Investigation of these factors as potential confounders or moderators was beyond the scope of this pilot study. Therefore, future larger scale studies will be necessary to investigate the effect of these variables on synovial fluid immune cell profiles following joint injury.

Of the CD4 subsets, Th1, Th2, and Th17 were the dominant populations present in the injured synovial fluid. Similarly, Lurati et al. [33] analyzed blood samples from RA, OA, and healthy subjects. They found that RA samples had the highest percentage of both CD4 T cells and Th17 cells followed by OA samples. However, there were no differences in Th1 or Th2 cell percentages between subject groups. Th17 cells produce IL-17, which causes synovial fibroblasts, chondrocytes, macrophages, and osteoclasts to elicit a cascade that promotes inflammation, cartilage degradation, and changes in bone metabolism [41]. Additionally, Rosshirt et al. also found higher numbers of CD4 T cells, favoring Th1 cell activation, in the synovial fluid of end-stage OA subjects compared to their peripheral blood, indicating localized joint inflammation [35]. Yang et al. also found that CCR4+CCR6+ Th cells (encompasses Th17 and Th22 cells) are directly correlated with anterior knee laxity in ACL-reconstructed human subjects [54]. Prior work has shown that naïve T cells can be differentiated into Th1 or Th17 cells by senescent cells, which are involved in age-related primary OA [53]. Recently, PTOA following tibial plateau fracture was found to be more advanced in T-cell deficient mice than in control mice [55]. The T cell deficiency eliminates all T cell subsets, both inflammatory and regulatory, and it is currently unknown whether the increased PTOA is due to a lack of regulatory T cells. Our results demonstrate the presence of regulatory T cells in the synovial fluid that can modulate inflammation. As well, the present study included ACL and meniscus injuries, while other joint injuries may lead to different immune cell profiles. Thus, there is a need to further profile-specific phenotypes of PTOA in addition to different types of arthritis.

With regard to CD8 T cells, there is limited in vivo human data characterizing their profile following joint injury. In a previous animal study, OA was induced via ACL transection causing increased activation of CD8 T cells and elevated tissue inhibitor of metalloproteinase-1 in the synovium and splenocytes, which correlated with increased cartilage degeneration [56]. In RA, there have been a few studies showing that an increased apoptotic-epitope of CD8 T cells was predictive of non-responsiveness to anti-TNF-⍺ therapies [57]. Additionally, in synovial fluid, effector memory CD8 T cells were found to be increased in RA subjects compared to healthy controls [58]. Given that similar T helper cells and CD8 T cells are found in subjects with more advanced OA and RA, our findings suggest that these cells present after ACL and meniscus injuries may be contributing to the development of PTOA. Furthermore, the similar profiles of CD4 and CD8 subsets following these knee injuries are consistent with the similar phenotype of PTOA following meniscus and ACL injuries. On the other hand, this was a pilot study and may not be powered to detect differences by injury type. In addition, this study included patients with a wide range of time from injury to knee surgery; therefore, in future larger scale studies, it will be important to investigate the time course of T cell profiles after ACL and meniscus injuries.

## Conclusion

In conclusion, at the time of surgery, the localized synovial fluid immune response following ACL and meniscus injuries shows a T cell-predominant immune profile in ACL, meniscus, and ACL+meniscus-injured knees. Among these injured knees, the percentage of CD4 T cells was significantly higher in the ACL- than the ACL+meniscus-injured knees. While there were no detectable differences in T cell subsets by injury type, Th1 and Th17 activation was favored among the CD4 T cells, which is supported by their active roles in OA. Overall, the findings of the present study describe both broad and T cell-specific immune profiles of human synovial fluid from ACL and/or meniscus-injured and normal knees. Future studies will focus on longitudinal studies to better understand the kinetics of T cell differentiation and effector function in relation to the development of PTOA.

## Data Availability

Data generated and analyzed during this study are available from the corresponding author on reasonable request.

## Abbreviations

ACL: Anterior cruciate ligament
COMP: Cartilage oligomeric matrix protein
IFN: Interferon
IL: Interleukin
MIP: Macrophage inhibitory protein
MMP: Matrix metalloproteinases
NK: Natural killer cells
PFC: Polychromatic flow cytometry
PGE2: Prostaglandin E2
PTOA: Post-traumatic osteoarthritis
OA: Osteoarthritis
RA: Rheumatoid arthritis
Tfh: T follicular helper cells
TNF: Tumor necrosis factor
Tregs: Regulatory T cells
